# The effects of painless nerve growth factor on human microglia polarization

**DOI:** 10.3389/fncel.2022.969058

**Published:** 2022-10-21

**Authors:** Lucia Lisi, Silvia Marinelli, Gabriella Maria Pia Ciotti, Michela Pizzoferrato, Federica Palmerio, Marta Chiavari, Antonino Cattaneo, Pierluigi Navarra

**Affiliations:** ^1^Section of Pharmacology, Department of Healthcare Surveillance and Bioethics, Catholic University Medical School, Fondazione Policlinico Universitario A. Gemelli-IRCCS, Rome, Italy; ^2^European Brain Research Institute-Fondazione Rita Levi Montalcini, Rome, Italy; ^3^Bio@SNS Laboratory, Scuola Normale Superiore, Pisa, Italy

**Keywords:** microglia, NGF (nerve growth factor), TrkA (tropomyosin receptor kinase), p75NTR, painless NGF

## Abstract

Previous studies in the rat suggest that microglial cells represent a potential druggable target for nerve growth factor (NGF) in the brain. The painless human Nerve Growth Factor (hNGFp) is a recombinant mutated form of human nerve growth factor (hNGF) that shows identical neurotrophic and neuroprotective properties of wild-type NGF but displays at least 10-fold lower algogenic activity. From the pharmacological point of view, hNGFp is a biased tropomyosin receptor kinase A (TrkA) agonist and displays a significantly lower affinity for the p75 neurotrophin receptor (p75NTR). This study aimed to evaluate the expression of TrkA and p75NTR NGF receptors in two different human microglia cell lines, and to investigate the effects of hNGFp and wild-type NGF (NGF) on L-arginine metabolism, taken as a marker of microglia polarization. Both NGF receptors are expressed in human microglia cell lines and are effective in transducing signals triggered by NGF and hNGFp. The latter and, to a lesser extent, NGF inhibit cytokine-stimulated inducible nitric oxide synthase (iNOS) expression and nitric oxide (NO) production in these cells. Conversely NGF but not hNGFp stimulates arginase-mediated urea production.

## Introduction

The painless human Nerve Growth Factor (hNGFp) is a recombinant mutated form of human nerve growth factor (hNGF), with two different mutations: glutamic acid replacing arginine in position 100, and serine [present in mouse nerve growth factor (NGF)] replacing proline in position 61 (Covaceuszach et al., 2010; Cattaneo and Capsoni, 2019). The first mutation reduces the binding affinity of hNGF to p75 neurotrophin receptor (p75NTR, mainly involved in apoptosis), without modifying the affinity for the tropomyosin receptor kinase A (TrkA) receptor, which mediates neuronal survival (Covaceuszach et al., 2010). The second mutation allows the selective detection of mutated NGF in biological fluids using a specific monoclonal antibody, referred to as 4GA (Covaceuszach et al., 2009). *In vivo*, hNGFp shows identical neurotrophic and neuroprotective properties of wild-type NGF (Capsoni et al., 2011; Severini et al., 2017) but displays at least 10-fold lower algogenic activity (Capsoni et al., 2012; Malerba et al., 2015).

The use of wild-type NGF in the clinical setting to investigate human NGF as a treatment for diabetic polyneuropathy and peripheral neuropathies in HIV led to trial discontinuation, after reports of a potent dose-dependent hyperalgesia, such as back pain, injection site hyperalgesia, and severe myalgia (Apfel, 1999a,b, 2002).

A large body of preclinical work showed that NGF exerts algogenic activity on the sensory neurons of dorsal root ganglia and spinal cord, where it modulates the transmission of pain signals, and activates the release of inflammatory mediators from inflammatory cells (Pezet and McMahon, 2006; Lewin and Nykjaer, 2014; Bannister et al., 2017).

Beyond the well-established role as a neurotrophic factor, NGF has been also identified as a regulatory factor in many non-neuronal cell types expressing NGF receptors, including fibroblasts, epithelial, endothelial, and immune cells (Minnone et al., 2017; Gostynska et al., 2020). NGF is produced by astrocytes and microglia, where NGF expression is markedly upregulated by local tissue injury, inflammation, and cytokines (Pöyhönen et al., 2019). In mouse cortex, TrkA is expressed in microglia, whereas p75NTR is expressed at very low levels in these cells (Rizzi et al., 2018). Both receptors were also observed in the cortex of phosphate buffer salt (PBS)-treated 5xFAD mice, an animal model of Alzheimer’s disease (Capsoni et al., 2017; Cattaneo and Capsoni, 2019). In this context, the effects of hNGFp on glial cells were found to be associated to a potent and broad neuroprotection in the 5xFAD aggressive neurodegeneration model. The effects of hNGFp appeared to be mediated by the chemokine CXCL12 (Capsoni et al., 2017). Rizzi et al. (2018) also showed that NGF acts on mouse microglia by polarizing cells toward a neuroprotective and anti-inflammatory phenotype. The above evidence from animal models shows that microglia represent a target cell of NGF in the brain. Thus, the relationship existing between hNGFp and inflammatory chemokines and cytokines at microglial level can be foreseen as a novel therapeutic target in the treatment of neurodegenerative diseases (Rizzi et al., 2018). However, evidence is lacking about the presence and role of NGF and its receptors in human microglia.

Here, for the first time, we investigated the expression of NGF receptors and the functional role of hNGFp in human microglial cells. In particular, this study aimed at evaluating the expression of TrkA and p75NTR in two different human microglial cell lines and to investigate the effects of hNGFp on inflammatory parameters. Notably, we found that both p75NTR and TrkA are expressed in human microglia. In addition, the effects of the painless hNGFp were compared to those of recombinant human NGF.

## Materials and methods

Cell culture reagents [Dulbecco’s modified Eagle’s medium (DMEM) and Fetal calf serum (FCS)] were from Invitrogen Corporation (Paisley, Scotland). Antibiotics were from Biochrom AG (Berlin, Germany). The human recombinant interleukin-1β (IL-1β), human interferon γ (IFNγ), recombinant human tumor necrosis factor α (TNFα), wild-type hNGF were purchased from R&D System (Minneapolis, MN, USA). The mixture of cytokine (TNFα-IFNγ-IL1β) will be referred as TII. The hNGFp was kindly supplied by Chiesi Farmaceutici.

### Cell cultures

The human microglia CHME5 cell line (CHME-5; https://www.cellosaurus.org/CVCL_5J53) was kindly provided by Professor Pierre Talbot (INRS-Institut Armand-Frappier, Laboratoire de Neuroimmunovirologie, Montréal, QC, Canada). CHME5 cells were grown in DMEM media containing 10% FCS and antibiotics. Experimental conditions were reached with DMEM at low concentration of FCS (1%), and cells were split at the 80% of the confluence.

Immortalized Human Microglia—SV40 (Imhu; Cat.No.: T0251) was purchased from Applied Biological Materials Inc. (Richmond, QC, Canada). Imhu was grown in Prigrow III media containing 10% FCS and antibiotics in Applied Biological Materials Inc. (Richmond, QC, Canada) PriCoat™ T25 flasks (Cat. No.: G299) and seeded at 40,000 cells per cm^2^ density. Experimental conditions were reached with DMEM-F12 at a low concentration of FCS (1%) in handmade Corning T25 coated flask with collagen I at 0.1 mg/mL (Cat. No.: A10483-01—Gibco Life Technologies Corporation, Grand Island, NY, USA) concentration (Chiavari et al., 2019).

### Immunostaining

Cover glasses of 13 mm of diameter were coated with Collagen I 0.1 mg/mL or Poli-L-lysine 50 μg/mL (Cat. No.: A-003-E—Merck Millipore, Burlington, MA, USA) for 1 h at 37°C. Thereafter, three washes in phosphate buffer salt (PBS) with calcium and magnesium were carried out before seeding the cells at a concentration of 50,000–100,000 cells per well. After 24 h from the treatment, cells were blocked with 4% PAF in PBS with calcium and magnesium for 20 min at room temperature. After three washes in PBS with calcium and magnesium, cells were blocked and permeabilized in PBS + 0.3% Triton X-100 + 5% Normal Donkey Serum (NDS) for 30 min at room temperature and incubated in the presence of primary antibody (specific concentrations in PBS + 0.1% Triton X-100 + 5% NDS). The incubation time was 2 h for Phalloidin 1:400 (Cat. No.: P1951—Sigma-Aldrich, St.Louis, MO, USA) and overnight 4°C for other primary antibodies. Primary antibodies were incubated in PBS + 0.1% Triton X-100 + 5% NDS. The following primary antibodies were used: guinea pig anti-iba1 1:500 (Cat. No. 234 004, SYSY); monoclonal mouse anti-TrkA, MNAC13, 1:300 (Cattaneo et al., 1999) and rat anti-p75 1:500 (SC-58567, SANTACRUZ). Afterward, cells were incubated in secondary antibodies dissolved in PBS + 0.1% Triton X-100 + 5% NDS, for 1 h 30′, at +4°C. DAPI 1:4000 was applied for 5 min in the second rinse, dissolved in PBS + 0.1% Triton X-10. The following secondary antibodies were employed: goat anti-guinea pig Alexa 647 (A21450, Thermo Fisher), goat anti-mouse Alexa 555 (A32727, Thermo Fisher), and donkey anti-Rat Alexa 488 (A-21208, Invitrogen). For the negative control (only secondary Abs) the incubation was performed only with PBS + 0.1% Triton+5% NDS.

For quantitative analysis, two blinded examiners counted the number of nuclei (DAPI + cells), in four randomly different areas of the glass. The average of four counts per each treatment was reported.

### Confocal microscopy

Analysis of the samples was performed using a X-light V2 confocal FLUO SPIN SIM (Crisel Instruments, Italy) with a fiber-coupled LED lightsource (Lumencor, US), an IX73 microscope (Olympus, Japan) with *x* axis motorization and an epifluorescence white lightsource Lumen 200 (Prior Scientific, US) equipped with LEDs Light Source with bandpass excitation filters of 460–490 and 535–600 nnm (Chroma Technology, US), controlled by MetaMorph acquisition and analysis software (Molecular Devices, US). Confocal images were acquired with a 40x/1.35 NA UApo, 63x/1.35 UplanSApo, and 100x/1.45 PlanApo oil objectives (Nikon, Japan). Z-stacks were obtained with a z-step size of 0.5 μm and with a format of 1,920 × 1,080 pixels. For each sample field all the channels (green: Alexa Fluo 488; red: Alexa Fluo 555; far red Alexa Fluo 647, DAPI) were simultaneously acquired. The SIM raw data with 16-bit depth was computationally reconstructed, channel specifically, using the Metamorph software package while post-production analysis was performed using FIJI.

### Nitrite assay

Inducible nitric oxide synthase (iNOS) activity was assessed indirectly by measuring nitrite accumulation in the incubation media. Briefly, an aliquot of the cell culture media (80 μL) was mixed with 40 μL Griess Reagent (Merck) and the absorbance measured at 550 nm in a spectrophotometric microplate reader (PerkinElmer, Waltham, MA, USA). A standard curve was generated during each assay in the range of concentrations 0–100 μM using NaNO2 (Merck) as standard. In this range, the detection of NaNO2 resulted linear and the minimum detectable concentration was 3.12 μM. In the absence of stimuli, basal levels of nitrites were below the detection limit of the assay at all the time points analyzed. Nitric oxide levels were normalized with the protein content determined by Bradford method (Bio-Rad Laboratories, Hercules, CA, USA) using bovine serum albumin (BSA) as standard.

### Urea assay

The levels of urea produced by CHME5 and Imhu cells were detected by the QuantiChrom Urea Assay kit (BIOassay System, Hayward, CA, USA), used according to the manufacturer’s instructions. Briefly, an aliquot of cell culture media (50 μL) was mixed with 200 μL Urea Reagent (Bioassay system) and the absorbance measured at 430 nm in a spectrophotometric microplate reader (PerkinElmer). A standard curve was generated during each assay in the range of concentrations 0–100 μg/ml using urea as standard. In this range, the detection of urea resulted linear, and the minimum detectable concentration was 3.12 μg/mL. Protein content in each sample was determined by Bradford method (Bio-Rad Laboratories) using BSA as standard.

### Real-time quantitative RT-PCR analysis

Total RNA from cell lines was extracted using the Trizol reagent protocol following the manufacturer’s instructions. RNA concentration was measured using the Qubit™ RNA HS Assay Kit (Thermo Fisher Scientific). One-μg aliquots of RNA were converted to cDNA using random hexamer primers. Quantitative changes in mRNA levels were estimated by real-time quantitative RT-PCR (qRT-PCR) using the following conditions: 35 cycles of denaturation at 95°C for 20 s; annealing and extension at 60°C for 20 s; using the Brilliant III Ultra-Fast SYBR^®^ Green QPCR Master Mix (Stratagene, San Diego, CA, USA). PCR reactions were carried out in a 20-μL reaction volume in AriaMX real time PCR machine (Agilent). Gene expression was evaluated using the following primers: iNOS forward primers CTGCATGGAACAGTATAAGGCAAAC, reverse primers CAGACAGTTTCTGGTCGATGTCA TGA (product length 230) and tubulin forward primers CCC TCG CCA TGG TAA ATA CAT, reverse primers ACT GGA TGG TAC GCT TGG TCT (product length 110). Relative mRNA concentrations were calculated from the take-off point of reactions (threshold cycle, Ct) using the comparative quantitation method provided by AriaMX software (Agilent Aria v1.5) and based upon the ^–^^Δ^ ^Δ^ Ct method. Ct values for Tubulin expression served as a normalizing signal.

### Western immunoblot analysis

CHME-5 were treated with NGF 10 ng/ml and hNGFp 10 ng/ml for 2 h. All the treatments were prepared in medium containing 1% FBS and 1% Penicillin–Streptomycin. At the end of experiment cells were scraped in PBS without Ca^2+^ and Mg^2^ and centrifuged at 1,100 rpm for 5 min. Then, cells were lysed in RIPA buffer [1 mM EDTA (Cat. No.: E7889), 150 mM NaCl (Cat. No.: S9888), 1% igepal (Cat. No.: I3021), 0.5% sodium deoxycholate (Cat. No.: D-6750), 50 mM Tris–HCl, pH 8.0 (Cat.No.: T-3038) (Sigma-Aldrich, St.Louis, MO, USA), and 0.1% sodium dodecyl sulfate, SDS, (Cat. No.:1610416—Bio-Rad, Hercules, CA, USA)] containing protease inhibitor cocktail diluted 1:250 (Cat. No: P8340—Sigma–Aldrich, St.Louis, MO, USA). Eventually, cells were centrifuged at 4°C for 10 min at 13,000 rpm and stored at −80°C. Bradford protein assay was performed to evaluate the total protein amount for each treatment. Briefly, 80 μg of proteins/sample were mixed with 4×Bolt™ LDS Sample Buffer (Cat. No.: B0007—Novex, Carlsbad, CA, USA) and 10×Bolt™ Sample Reducing Agent (Cat. No.: B0009—Novex, Carlsbad, CA, USA), boiled for 5 min at 95°C and, eventually, loaded in a precast gel (NuPAGE™ 4–12%, Bis-Tris, 1.0–1.5 mm, Mini Protein Gels, Invitrogen). After electrophoresis, carried out at 200 V for 5 min and at 150 V for 80 min, proteins were transferred to a PVDF membrane (Invitrogen, Carlsbad, CA, USA) using iBlot™ 2 Gel Transfer Device (Invitrogen, Carlsbad, CA, USA) and different antibodies were tested. All the primary antibodies were prepared in Flex Solution (iBind™ Flex Solution Kit, Invitrogen, Carlsbad, CA, USA) and were incubated for 2 h at room temperature or overnight at 4°C with gentle shaking, as shown in Table 1. The day after, the primary antibody was removed, and the membrane was washed three times with TBS-T. After that, the membrane was incubated for 1 h with the secondary antibody, dissolved in Flex Solution (iBind™ Flex Solution Kit, Invitrogen, Carlsbad, CA, USA). Following three washes in TBS-T, bands were detected by chemiluminescence (ChemiDoc™ XRS, Bio-Rad), rinsing the membrane with ECL reagents (SuperSignal™ West Pico PLUS Chemiluminescent Substrate, Thermo Scientific™, Rockford, IL, USA, and Pierce™ ECL Western Blotting Substrate). Primary and secondary antibodies, the related dilutions and the incubation time are reported in the table below.

**TABLE 1:** Antibody information used for western immunoblotting analysis.

Antibody	Dilution	Producer	Incubation time and temperature conditions
anti p- AKT ser 473 XP	*1:1000*	Cell signaling	2 h, room temperature
anti tot AKT	*1:1000*	Thermo Fisher Scientific	2 h, room temperature
anti p- Creb	*1:1000*	Cell signaling	overnight, 4°C
anti β-actin	*1:1000*	Sigma	2 h, room temperature
anti p-MEK 1/2	*1:1000*	Cell signaling	overnight, 4°C
anti total MEK 1/2	*1:1000*	Cell signaling	overnight, 4°C
anti IkB α	*1:1000*	Santa Cruz	2 h, room temperature
Anti-mouse	*1:3000*	Sigma	1 h, room temperature
Anti-rabbit	*1:15000*	Jackson ImmunoResearch	1 h, room temperature

### Statistical analyses

Data were described as means ± Standard Deviation (SD) or standard error of the mean (SEM) as indicated in figure legends. Statistical analysis of the differences between paired groups was performed by Student’s *t*-test. For multiple comparisons ANOVA analysis, followed by Sidak’s post-test, was used. Statistical significance was determined at α = 0.05 level. Differences were considered statistically significant when *p* < 0.05. Statistical analysis was performed with GraphPad software Prism version 7.04 (GraphPad Software, San Diego, CA, USA).

## Results

**FIGURE 1:**
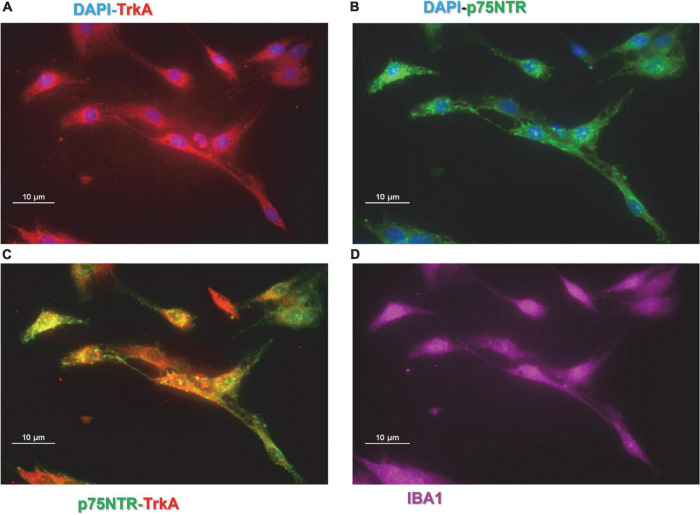
Expression of nerve growth factor (NGF) receptors in CHME-5 cell lines. **(A)** Tropomyosin receptor kinase A (TrkA) positive cells. **(B)** p75 neurotrophin receptor (P75NTR) positive cells. **(C)** Co-localization of P75NTR and TrkA on CHME-5. **(D)** IBA positive cells. Magnitude 60X; scale bar 10 μm.

**FIGURE 2:**
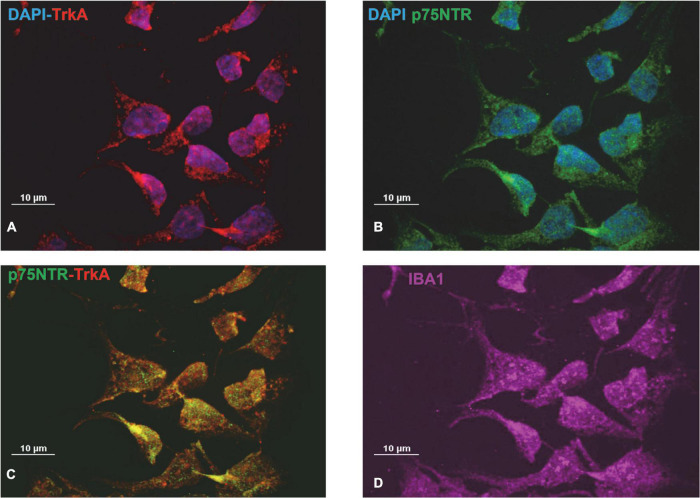
Expression of nerve growth factor (NGF) receptors in Imhu cell lines. **(A)** Tropomyosin receptor kinase A (TrkA) positive cells. **(B)** p75 neurotrophin receptor (P75NTR) positive cells. **(C)** Co-localization of P75TNTR and TrkA on Imhu. **(D)** IBA positive cells. Magnitude 60X; scale bar 10 μm.

Preliminary experiments were carried out to investigate whether CHME-5 and Imhu cells express p75NTR and TrkA receptor. Immunofluorescence experiments were carried out under basal condition. As expected, both cell lines are IBA^+^ (Figures 1D, 2D), the latter being used as microglia/macrophage marker. Both NGF receptors, p75NTR (Figures 1A, 2A) and TrkA (Figures 1B, 2B) are present in CHME-5 and Imhu lines. In CHME-5, TrkA presents as a diffuse cytoplasmatic receptor, whereas p75NTR is represented as more pinpointed receptor, in both nuclei and cytoplasm. In Imhu cells, both TrkA and p75NTR show as pinpointed receptors, in both nuclei and cytoplasm. The different localization of receptors is also shown in Figures 1C, 2C, where the co-localization of the two receptors is observed. In particular, in the cytoplasm of CHME-5 there is a co-localization of p75NTR and TrkA (orange color in Figure 1C), whereas in the Imhu the co-localization is present in both nuclei and cytoplasm (orange color in Figure 2C).

**FIGURE 3:**
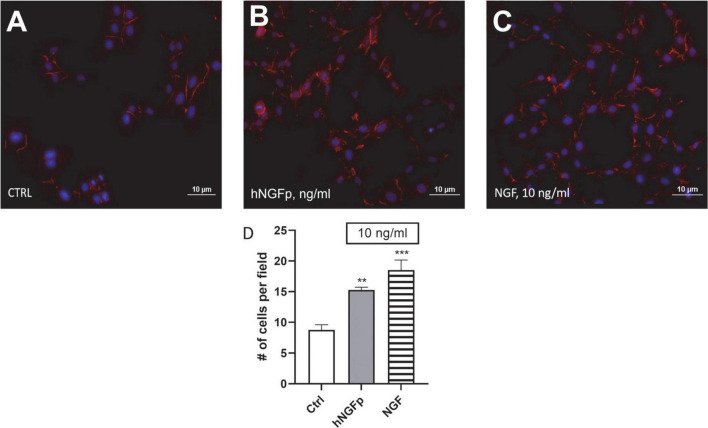
Evaluation of CHME-5 morphology after nerve growth factor (NGF) treatments. The cells were analyzed under basal condition **(A)**, after 24 h 10 ng/ml of painless human Nerve Growth Factor (hNGFp) treatment **(B)** and after 24 h 10 ng/ml wild type NGF treatment **(C)**: Magnitude 20X. Quantification of cell number elicited by NGF treatment **(D)**. One-way ANOVA analysis followed by Bonferroni’s post-test was carried out *F*(2, 9) = 19.94; *P* = 0.0005. The experiment was repeated three times with similar results. **p < 0.01 and ***p < 0.001.

To verify the functional activity of NGF receptors, experiments were carried out to investigate cell morphology; moreover, different proteins were studied through western blot analysis. Phalloidin immunostaining analyses showed that both hNGFp and wild type NGF modify the morphology of microglia from sparse single cells to more dense cell networks (Figure 3). In addition, both hNGFp and NGF were able to significantly increase the number of CHME-5 cells (Figure 3D). In particular, the number of cells was increased by about 40 and 50% after exposure to hNGFp and NGF, respectively.

**FIGURE 4:**
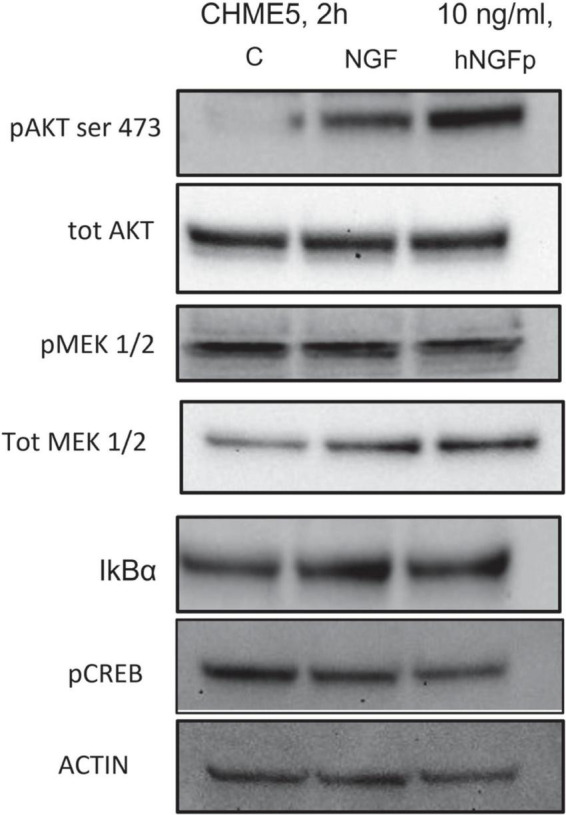
Evaluation of tropomyosin receptor kinase A (TrkA) and p75NTR pathway in CHME-5 cells treated for 2 h with nerve growth factor (NGF) isoforms. Treatments: lanes 1, control; lane 2, 10 ng/ml NGF; lane 3, 10 ng/ml painless human Nerve Growth Factor (hNGFp). The following proteins were evaluated: pAKT (ser 473); tot AKT, p MEK1/2, tot MEK, IkBα, pCREB, and actin.

In order to investigate the signal pathways activated by NGFs downstream p75NTR and/or TrkA stimulation, different proteins were studied (Figure 4). Two-hour experiments were carried out. Both wild type NGF and hNGFp were able to significant increase the phosphorylation of ATK (ser 473), taken as direct effector of TrkA activation. In addition, after 2 h of NGF exposure, the phosphorylation of MEK1/2 was not modified, but an increase of total MEK level was observed. Finally, a slight reduction in pCREB expression was also induced by both NGF and hNGFp. AKT, MEK1/2 and CREB are downstream TrkA factors, whereas NFkB is a downstream transcription factor of p75NTR activation. Figure 4 shows an increase in IkBα protein (an inhibitor of NFkB) after exposure to NGF, whereas hNGFp was not able to modify IkBα levels.

**FIGURE 5:**
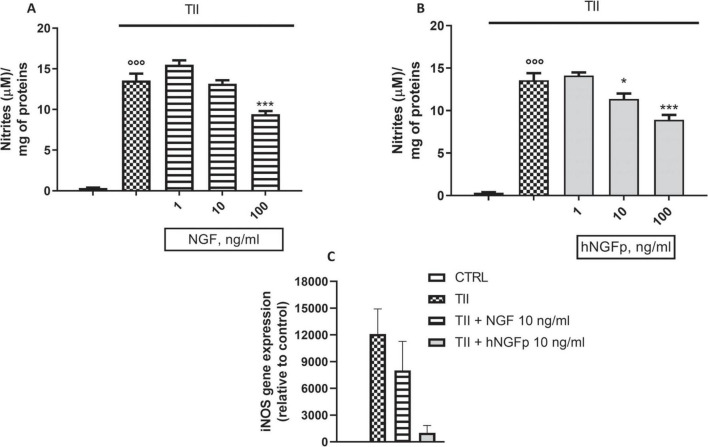
Effects on inducible nitric oxide synthase (iNOS) pathway after 48 h nerve growth factor (NGF) treatments. CHME-5 were treated with TII for 48 h. **(A)** The effects of 1–10 or 100 ng/ml of wild type Nerve Growth Factor (NGF) on nitrite production in CHME-5. **(B)** The effects of 1–10 or 100 ng/ml of painless human NGF on nitrite production in CHME-5. **(C)** iNOS expression after 24 h of treatment.°°°*p* < 0.001 vs. control, *p < 0.05 and ***p < 0.001 vs. TII. One-way ANOVA analysis followed by Bonferroni’s post-test was carried out. **(A)** *F*(4, 25) = 101.2; *P* < 0.0001. **(B)** *F*(4, 25) = 141.2; *P* < 0.0001. Data are expressed as mean ± SEM. The experiment was repeated four times with similar results.

Subsequent experiments were carried out to look at the effects of hNGFp and NGF on L-Arginine metabolism, in particular on the production of nitric oxide (NO) and urea. In CHME-5 cells TII, a standardized mixture of proinflammatory cytokines, elicits a huge increase in both iNOS expression and nitrite production after 48 h of exposure (Figure 5). Under this condition, both hNGFp and NGF (range dose 1–100 ng/ml) were able to counteract in a dose-dependent manner NO production (Figures 5A,B) and iNOS gene expression (Figure 5C) induced by the cytokine mixture. In particular, to get a reduction of nitrite production in the same order of magnitude, 100 ng/ml of NGF and 10 ng/ml of hNGFp (i.e., one order of magnitude less than NGF) had to be used. Such difference in potency was consistent with the analysis of iNOS gene expression after 24 h of treatment, which showed that hNGFp but not NGF, both given at 10 ng/ml, significantly inhibited cytokine-induced iNOS gene expression. No change was elicited by both NGF isoforms under baseline conditions, without TII stimuli (data not shown).

**FIGURE 6:**
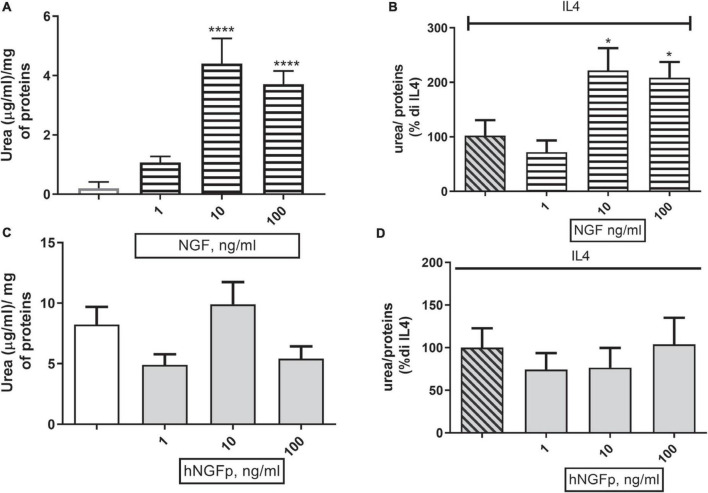
Effects on urea pathway after 48 h nerve growth factor (NGF) treatments in CHME-5. **(A,B)** The effects of 1–10 or 100 ng/ml of NGF on urea production under basal condition and in co-stimulation with IL4 respectively. **(C,D)** The effects of 1–10 or 100 ng/ml of painless human Nerve Growth Factor (hNGFp) on urea production under basal condition and in co-stimulation with IL4 respectively. *p < 0.05 and ****p < 0.0001 vs. IL4. One-way ANOVA analysis followed by Bonferroni’s post-test was carried out. **(A)** *F*(5, 30) = 16.54; *P* < 0.0001. **(B)** *F*(3, 19) = 6.368; *P* = 0.0036. Data are expressed as mean ± SEM. The experiment was repeated four times with similar results.

**FIGURE 7:**
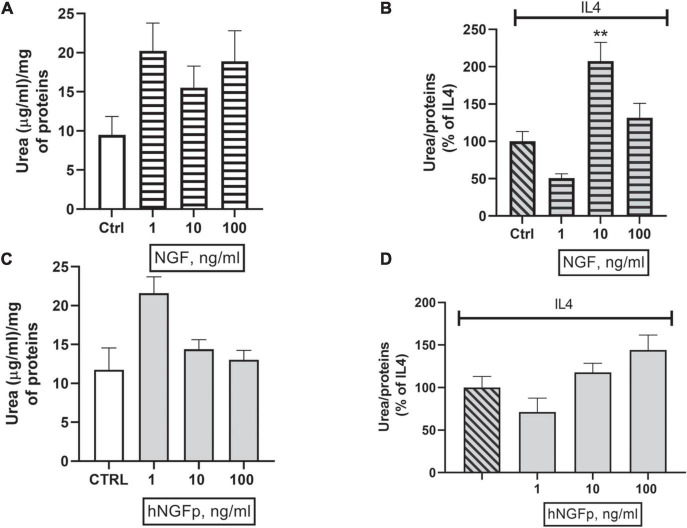
Effects on urea pathway after 48 h nerve growth factor (NGF) treatments in Imhu. **(A,B)** The effects of 1–10 or 100 ng/ml of wild type NGF on urea production under basal condition and in co-stimulation with IL4 respectively. **(C,D)** The effects of 1–10 or 100 ng/ml of painless human Nerve Growth Factor (hNGFp) on urea production under basal condition and in co-stimulation with IL4 respectively. **p < 0.01 vs. IL4. One-way ANOVA analysis followed by Bonferroni’s post-test was carried out. **(B)** *F*(3, 13) = 12.02; *P* = 0.0005. Data are expressed as mean ± SEM. The experiment was repeated two times with similar results.

Concerning urea production, 10 and 100 ng/ml of wild type NGF increase the production of urea both under basal conditions (Figure 6A) and after stimulation by IL-4 (Figure 6B) in 48-h experiments. At variance with wild-type NGF, hNGFp given in the range 1–100 ng/ml, had no effect whatsoever on urea production, either under basal conditions (Figure 6C) and after stimulation with IL-4 (Figure 6D). Urea production has been also studied in Imhu cells. Similar to CHME-5cells, wild type NGF was able to increase urea levels after 48 h of exposure in the presence of IL4 (Figure 7B), and tends to increase urea production under basal condition, however without reaching statistical significance (Figure 7A). Finally, like in CHME-5, no significant effect was observed after exposure of Imhu to hNGFp (range 1–100 ng/ml), neither in baseline conditions nor after stimulus (Figures 7C,D).

## Discussion

To the best of our knowledge, this is the first study showing that NGF receptors are expressed in human microglia cells and are effective in transducing signals triggered by NGF and its mutated form, hNGFp.

An important first finding was that both NGF receptors (p75NTR and TrkA) are expressed under basal conditions in two different human microglia lines. These findings in human cells confirm previous observations in other species (Meeker and Williams, 2015; Garcia et al., 2017; Rizzi et al., 2018). In addition, in human cell lines as in other species, TrkA signaling involve AKT, MEK, and CREB, whereas p75NTR signaling involve NFkB.

Previous studies in the mice point out microglia a potential druggable target for both NGF and hNGFp in the brain (Capsoni et al., 2017; Rizzi et al., 2018). Indeed, a new anti-neurodegenerative pathway, linking hNGFp to chemokines and cytokines at the level of microglia, was discovered. As such this new anti-neurodegenerative pathway extends the neuroprotective potential of hNGFp beyond the classical cholinergic paradigm, thereby widening the range of neurological diseases that become a possible target to explore the therapeutic efficacy of hNGFp (Capsoni and Cattaneo, 2022).

In the present study, we sought to investigate the effects of NGF and its painless analog on microglia activation. The two cell lines used show different characteristics between each other, which might prevent comparing some parameters (Zhang and Cui, 2021). In fact, CHME-5 cells do respond to pro-inflammatory stimuli by expressing iNOS and producing nitric oxide, whereas Imhu cells are not sensitive to standard stimulation by the cytokine mix (Chiavari et al., 2019).

Although seriously questioned, the traditional M1/M2 microglia classification is still widely used due to the existence of objective biomarkers and it is useful in identifying the microglia phenotype since genes, receptors, agents, and molecules can be distinguishing factors of a certain polarization phase (Ransohoff, 2016).

Hence, the microglia/macrophage phenotype, whether showing an activated, proinflammatory (M1) status, or -on the contrary, an anti-inflammatory (M2) profile, is defined by looking at the activated/resting condition of a large array of signaling pathways (Amici et al., 2017). Our group has previously focused on the analysis of L-arginine (L-ARG) metabolic pathways as a key player of microglia regulation (Lisi et al., 2014a,b, 2019; Laudati et al., 2017; Chiavari et al., 2020). In fact, L-ARG acts as a substrate for two different enzymes, arginase (ARG) and oxide nitric synthase (iNOS). L-ARG catabolism through iNOS produces NO and L-citrulline, whereas its catabolism *via* ARG yields urea and ornithine. During inflammation, exaggerated NO production through the iNOS pathway promotes nitrative stress, leading to neurodegeneration and apoptosis, while an increased ARG activity may lead to excessive ornithine production, which in turn may cause vascular damage and neural toxicity (Burrack and Morrison, 2014). Since the two enzymatic pathways share the same substrate, an excessive iNOS activity can possibly reduce the amounts of L-ARG yielding urea through the ARG pathway, and vice versa. Therefore, the iNOS pathway is thought to as a marker of proinflammatory microglia phenotype whereas the ARG pathway is taken as a marker of anti-inflammatory microglia phenotype.

In the present study on human microglia, wild type NGF was found to reduce the expression and production of the iNOS, while on the same time increasing the production of the ARG. These data are consistent with previous findings by Rizzi et al. (2018), showing an all-encompassing anti-inflammatory activity, reduced iNOS markers and in parallel increased ARG activity. Conversely, hNGFp was even more potent that NGF in reducing the M1 marker but it failed to increase the production of M2 marker. Taking into account the different profile of receptor activation displayed by NGF and hNGFp, the present findings would suggest that the selective inhibition of certain signaling pathways downstream to TrkA activation by hNGFp (Capsoni et al., 2011) prevents a fully drive the microglia toward an M2 phenotype although an identical overall binding affinity for TrkA is retained.

M2 phenotype is associated with the ability to produce anti-inflammatory and immune suppressive factors, including ARG-1, Ym1, CD36, urea as well as to up-regulate the cell surface markers CD163, CD204, and CD206 and the anti-inflammatory cytokines, such as IL-10 (Amici et al., 2017). In a fairly simplistic syllogism, the M2 phenotype is associated, at least *in vitro*, with pro-tumor effects. In human pathology, there is not a clear distinction between the two phenotypes. Microglial cells within a tumor parenchyma often display a complex pattern of phenotypes, showing up-regulation of both M1 and M2 markers, with the prevalence of one phenotype on the other possibly depending on the stage of disease (Lisi et al., 2017). In light of such apparent difficulty in applying the M1/M2 paradigm to the central nervous system, it has been postulated that the notion of stimulus-dependent microglia phenotype should substitute that of microglia polarization (Rivera et al., 2021). Based on this novel paradigm, we might also conclude that hNGFp has a clear, better defined anti-inflammatory profile compared to wild type NGF.

## Data availability statement

The raw data supporting the conclusions of this article will be made available by the authors, without undue reservation.

## Author contributions

LL planned the experiments, analyzed the data, and drafted the manuscript. SM analyzed the data on NGF receptors. MC and GC carried out experiments. FP carried out NGF receptor experiments. MP carried out NGF protein experiments. AC concepted the research. PN concepted the research, analyzed the data, and reviewed the manuscript. All authors contributed to the article and approved the submitted version.
